# Moxetumomab pasudotox-tdfk for relapsed/refractory hairy cell leukemia: a review of clinical considerations

**DOI:** 10.1007/s00280-019-03875-6

**Published:** 2019-05-27

**Authors:** Carmen F. Nobre, Matthew J. Newman, Anne DeLisa, Pauline Newman

**Affiliations:** Department of Pharmacy, The Johns Hopkins Hospital, Baltimore, MD 21287 USA

**Keywords:** Hairy cell leukemia, CD22, Immunotoxin, Moxetumomab pasudotox-tdfk

## Abstract

**Purpose:**

Hairy cell leukemia (HCL) is a rare mature B cell leukemia. Purine analogs are the mainstay of treatment of HCL, but relapse after purine analog therapy is common. Outcomes of treatment of relapsed/refractory HCL typically diminish with each successive line of therapy. Moxetumomab pasudotox-tdfk is a novel recombinant immunotoxin approved for the treatment of patients with relapsed/refractory HCL who have received at least two prior therapies, including a purine analog. This article reviews HCL treatment, focusing on moxetumomab pasudotox-tdfk, its place in therapy, considerations for preparation and administration, and strategies for prevention and management of toxicities.

**Methods:**

A literature search was conducted in the PubMed database from inception to January 2019, using the following terms: moxetumomab, hairy cell leukemia, relapsed/refractory hairy cell leukemia, immunotoxin, and CD22. The package insert and available posters and abstracts were also reviewed.

**Results:**

FDA approval of moxetumomab pasudotox-tdfk was based on a phase III single-arm, open-label trial in 80 patients. Treatment with moxetumomab pasudotox-tdfk yielded a durable complete response rate of 30% with a median duration of response that had not yet been reached at a median follow-up of 16.7 months. The objective response rate was 75% based on blinded independent central review. The most common adverse reactions were infusion-related reactions, edema, nausea, fatigue, headache, pyrexia and anemia. Serious adverse events include capillary leak syndrome and hemolytic uremic syndrome.

**Conclusions:**

Clinicians providing care for patients receiving moxetumomab pasudotox-tdfk should be aware of the strategies required for safe administration, including the management of serious adverse events.

## Description and epidemiology

Hairy cell leukemia (HCL) is a rare, chronic, mature B-cell malignancy occurring in an estimated 1100 patients annually in the United States [1]. The disease is characterized by the presence of lymphocytes with cytoplasmic projections appearing hair-like by microscopy, which infiltrate the bone marrow and spleen resulting in cytopenias, bone marrow fibrosis, and splenomegaly [2]. HCL comprises only 2% of all leukemias, the median age at diagnosis of HCL is 50 years, and the disease has a 4:1 male predominance [3]. Environmental exposures associated with farming, pesticides, and ionizing radiation may be risk factors [4–6]. Outcomes are typically positive, with 78% of patients alive at 15 years; overall survival is 96% when censoring for deaths unrelated to HCL [7]. However, relapses occur in about half of patients initially treated with a purine analog after 16 years of follow-up, and outcomes including rate of complete response and duration of relapse-free survival are diminished with each successive line of treatment [7]. The 2008 World Health Organization classification recognized the classic form of HCL as distinct from the rarer variant form, which accounts for 10–20% of all HCL cases [8]. HCL variant is characterized by a poorer response to purine analog monotherapy, increased lymphocytosis, and cytopenias without monocytopenia, as compared with the classic form [9].

## Pathophysiology

Malignant cells infiltrate the bone marrow in nearly all patients with HCL [10]. Other affected organs include the spleen, liver, and lymph nodes [11]. Flow cytometry on peripheral blood or bone marrow aspirate in HCL will be positive for CD11c, CD19, CD20, CD22, CD25, CD103, CD123, cyclin D1, annexin A1, and CD200, and negative for CD5 and CD10 [12]. HCL variant is negative for CD25, CD123, and annexin A1 [12]. The BRAF V600E mutation is present in most cases of the classic form of HCL but absent in the variant [13]. Molecular analysis for the IGHV4-34 rearrangement may be employed. HCL expressing the IGHV4-34 rearrangement shares clinical features of the variant (including poorer response to purine analogs and poorer prognosis) and lacks BRAF mutation, but may be classified as classic HCL by immunophenotype [8, 11].

## Clinical characteristics

Patients with HCL present most commonly with fatigue, recurrent infections, and symptomatic or asymptomatic splenomegaly, with other symptoms including weakness, fevers, night sweats, weight loss, and bruising [8, 11]. Initial treatment of HCL is indicated in the presence of symptomatic disease, or cytopenias (hemoglobin < 11 g/dL, platelets < 100,000/µL and/or absolute neutrophil count [ANC] < 1000/µL), progressive lymphocytosis, or lymphadenopathy [14]. If the patient is asymptomatic, a reasonable approach is close observation with initial treatment upon development of symptoms [11].

## Review of treatment

Historically, HCL was managed with interferon and splenectomy [2]. These treatment approaches have been largely replaced by the purine analogs, pentostatin or cladribine. An early Eastern Cooperative Oncology Group study of pentostatin in untreated and previously treated HCL (including splenectomy, chemotherapy, or both) demonstrated a response rate of 96% and a complete response (CR) rate of 59% [15]. A randomized trial of pentostatin versus interferon alfa-2a in newly diagnosed HCL resulted in improved CR rates with pentostatin (76% vs. 11%, respectively), with improvements in response rate and relapse-free survival [16]. In a more recent long-term follow-up study of patients who received pentostatin, estimated survival rates were 90% at 5 years and 81% at 10 years, with two of 40 deaths attributed to HCL during the follow-up period [17].

Outcomes of treatment with cladribine in patients with HCL are described in Table 1. The majority of patients attain a CR, and, although overall survival is prolonged, a significant proportion will relapse and require additional therapy.

**Table 1:** Outcomes after initial treatment with cladribine

Study	Patients, *n*	Dosing	CR, %	PR, %	Relapse	OS
Estey et al. [53]	46	4 mg/m^2^ daily CIV for 7 days	78	11	One patient at 71 weeks (median follow-up 37 weeks)	Not evaluated
Saven et al. [54]	349	0.1 mg/kg daily CIV for 7 days	91	7	26% at median 29 months; 62% achieved CR with retreatment	96% at 48 months
Goodman et al. [18]	207	0.1 mg/kg daily CIV for 7 days	95	5	37% at median 42 months	97% at 108 months
Chadha et al. [55]	86	0.1 mg/kg daily CIV for 7 days	79	21	36% at median 9.7 years; 52% achieved CR with retreatment	87% at 12 years

*CIV* continuous intravenous infusion*, CR* complete response, *OS* overall survival, *PR* partial response

A retrospective review of 233 patients with HCL did not find any clinically relevant differences in outcomes between those treated with pentostatin and those treated with cladribine, including CR rate (82% vs. 76%, not significant [NS]), and the two are considered interchangeable for first- and second-line therapy [7]. The purine analogs are associated with profound neutropenia, reduction in CD4 + T cells, and resultant opportunistic infections [14].

For patients achieving a CR (hemoglobin > 11 g/dL without transfusion, platelets > 100,000/µL, ANC > 1500/µL, regression of splenomegaly on examination, and absence of morphologic HCL on peripheral blood smear and in bone marrow), observation is employed to detect relapse. Treatment options for patients relapsing 2 years or more after achieving a CR include retreatment with the original agent or the alternative purine analog with or without the addition of rituximab. Of patients with relapsed disease who were retreated with cladribine, the rate of second CR was 75% with a median response of 35 months [18]. Single-agent rituximab may be considered for those unable to tolerate a purine analog. If relapse occurs within 2 years, or a suboptimal response (less than a CR) is achieved with initial therapy, recommendations include a clinical trial, the alternative purine analog with or without rituximab, interferon alfa, rituximab monotherapy, or vemurafenib. In the setting of progression after second-line treatment, options prior to the Food and Drug Administration (FDA) approval of moxetumomab pasudotox-tdfk included a clinical trial, vemurafenib with or without rituximab, or ibrutinib [11].

Two smaller studies established the combination of rituximab and a purine analog for relapsed HCL. In the first, all 14 patients achieved CR and were alive at 5 years’ follow-up [19]. A second retrospective study resulted in a CR rate of 89%, which was maintained at a median follow-up of 36 months [20]. Rituximab monotherapy is somewhat less active than the combination of rituximab and a purine analog, with CRs ranging from 10 to 53% [21–24]. It is recommended mainly for patients unable to receive a purine analog [11].

Newer oral agents have been used effectively in relapsed/refractory (R/R) HCL. Vemurafenib has resulted in an overall response rate of 100% and a CR in 42% of patients at 12 weeks [25]. At 1 year, progression-free survival was 73%, and overall survival was 91%. Additionally, median relapse-free survival was 9 months in responders and 19 months for those with a CR. Toxicities included rash, arthralgias or arthritis, pyrexia, and elevated bilirubin. The addition of rituximab to vemurafenib yielded an observed CR rate of 100% in 25 evaluable patients, with 70% of patients achieving CR after 4 weeks of treatment [26]. Ibrutinib has been evaluated mainly in patients with relapsed classical and variant HCL previously treated with a purine analog. The objective response rate was 46% and more common in patients with classical HCL [27]. Progression-free survival at 24 months was 79%. Toxicities included grade 3 or higher lymphopenia (21%), neutropenia (18%), thrombocytopenia (14%), and infections in 25% of patients.

The risk of relapse in HCL has been proposed using the following categories based on minimal residual disease (MRD): < 1% positive cells, low risk; 1–5% intermediate risk; > 5% higher risk [28]. In patients treated with cladribine followed by rituximab, MRD was undetectable in 92–94% [19, 29]. In patients treated with vemurafenib and rituximab, MRD was undetectable in 73% of evaluated patients [30].

Moxetumomab pasudotox-tdfk (LUMOXITI^®^, AstraZeneca Pharmaceuticals LP) is a novel recombinant CD22-directed cytotoxin targeting CD22, which has been newly approved for the treatment of patients with R/R HCL who have received at least two prior therapies, including at least one purine analog [31]. This is the first FDA-approved therapy for R/R HCL since cladribine in 1993. Clinicians should be aware of the preparation, administration, monitoring, and adverse-effect management strategies necessary to ensure safe and effective use of moxetumomab pasudotox-tdfk.

## Immunotoxins and CD22

Immunotoxins are useful in delivering targeted anticancer therapy. They are proteins that consist of a targeting portion, such as an antibody or growth factor, linked to a toxin. Toxins used in immunotoxin products are procured from bacteria, fungi, and plants, and most function by inhibiting protein synthesis. Commonly used bacterial toxins include diphtheria toxin and *Pseudomonas* exotoxin (PE) [32]. Recombinant immunotoxins contain an Fv fragment fused to a truncated protein toxin [33]. Agents tested in HCL include LMB-2 targeting CD25 and BL22/CAT-3888 targeting CD22. BL22/CAT-3888 consisted of disulfide-linked VH and VL chains of the murine anti-CD22 monoclonal antibody RFB4 fused to a truncated form of exotoxin A, PE38 [34, 35]. BL22/CAT-3888 served as the precursor to CAT-8015/moxetumomab pasudotox-tdfk.

CD22 is a B-lymphocyte lineage-restricted transmembrane protein that first emerges on the face of pre-B-cells and is fully expressed by differentiated IgM^+^, IgD^+^ B-cells [36–39]. The cytoplasmic domain of CD22 has six tyrosines as potential targets for phosphorylation, with regions related to the tyrosine-based inhibition motif [38, 39]. Tyrosine-based inhibition motifs are also substrates for Src family tyrosine kinases, and docking sites for SH2 domains, including SHP-1. Association with these inhibitory proteins results in negative regulation of B-cell receptor (BCR) signaling, thus leading to apoptosis of B-cells [40, 41]. CD22 is rapidly phosphorylated after BCR cross-linking. It has a basal half-life of about 8 h and is internalized in less than 1 h once it is bound by a ligand or cross-linked, and it does not recycle to the cell surface from the intracellular pool. Once endocytosed, CD22 is directed to the lysosome for degradation [39]. CD22 is an attractive target for immunotoxin therapy, as it is a B-cell antigen expressed particularly strongly in HCL [42].

Moxetumomab pasudotox-tdfk (CAT-8015) is a second-generation recombinant immunotoxin, with increased affinity compared with BL22 [43]. Affinity for CD22 was increased 14-fold by hot-spot mutagenesis [44]. Moxetumomab pasudotox-tdfk contains threonine–histidine–tryptophan instead of serine–serine–tyrosine in the antigen-binding site of the heavy chain [45]. It is formed of a murine immunoglobulin variable domain genetically fused to a truncated form of *Pseudomonas* exotoxin, PE38 [31, 46].

**Fig. 1:**
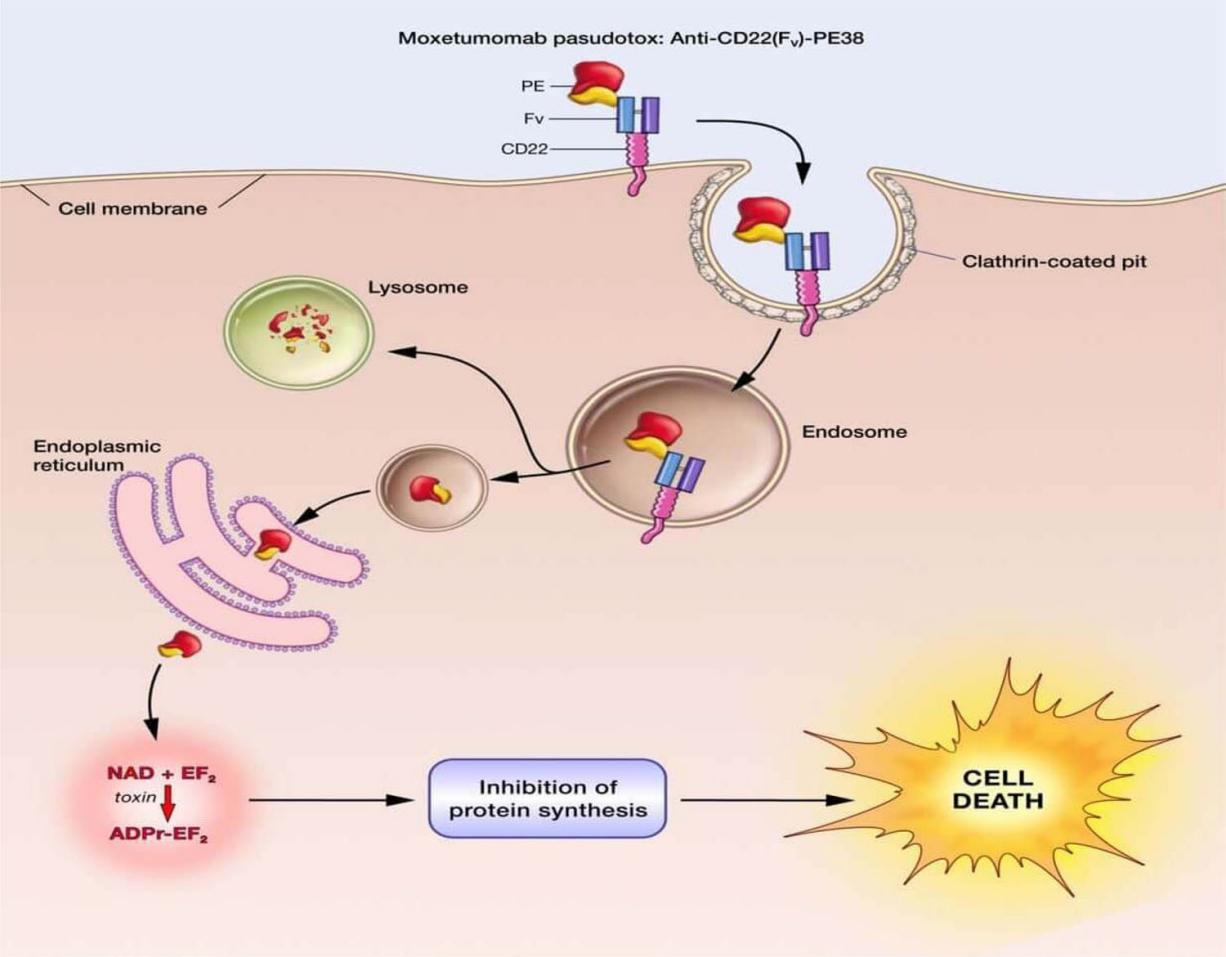
Moxetumomab pasudotox-tdfk mechanism of action. Reproduced from: https://www.cancer.gov/news-events/cancer-currents-blog/2018/moxetumomab-fda-hairy-cell-leukemia

After binding to CD22, moxetumomab pasudotox-tdfk is internalized, and the *Pseudomonas* exotoxin catalyzes inhibition of protein synthesis by ADP-ribosylation of elongation factor 2, resulting in apoptotic cell death (Fig. 1) [31].

## Clinical results

In an open-label phase 3 trial, 80 patients with R/R HCL with at least two prior therapies, including at least one purine analog, received moxetumomab pasudotox-tdfk 0.4 mg/kg/dose intravenously (IV) on days 1, 3, and 5 [47] of each 28 day cycle for up to six cycles or until disease progression or unacceptable toxicity [47]. At a median follow-up time of 16.7 months (range 2–49), durable CR, defined as the maintenance of hematologic remission (Hgb ≥ 11 g/dL, neutrophils ≥ 1500/mm^3^, and platelets ≥ 100,000/mm^3^ without transfusions or growth factor for at least 4 weeks) more than 180 days after IRC-assessed CR, was achieved in 30%, CR in 41%, PR in 34%, and objective response in 75%, with 80% achieving hematologic remission. Adverse effects included peripheral edema (39%), nausea (35%), fatigue (34%), and headache (33%). Serious adverse effects included hemolytic uremic syndrome (HUS) (7.5%) and capillary leak syndrome (CLS) (5%). Neutropenia and decreased neutrophil count occurred infrequently (5% and 7.5% of patients, respectively).

Results of 33 patients from the long-term follow-up dose escalation phase 1 study of patients with R/R HCL treated with moxetumomab pasudotox-tdfk 0.05 mg/kg/dose on days 1, 3, and 5 every 28 days were CR in 64% (median CR duration: 42.4 months) and overall response in 88% of patients [48]. Flow cytometric analysis of bone marrow aspirate was performed for MRD assessment. The median CR duration was longer in patients with MRD-negative status (*n* = 11) than in those with MRD-positive status (*n* = 9) (42.1 months vs. 13.5 months; *P* < 0.001). Most patients achieving MRD negativity remained in CR (10 patients), of whom 9 patients were without MRD at the time of study conclusion. Although the significance of MRD in HCL is not yet clearly defined, outcomes appear to be improved in patients achieving MRD negativity [49–51].

## Dosing and pharmacokinetics

The labeled dose and schedule of moxetumomab pasudotox-tdfk are 0.04 mg/kg/dose, using actual body weight, on days 1, 3, and 5 of a 28-day cycle. A change in dose should not be made during a cycle. Dose changes should only be made between cycles when a change in weight of greater than 10% is observed from the weight used to calculate the first dose of the first treatment cycle. Treatment should be continued for up to six cycles unless there is disease progression or severe toxicity including CLS or HUS. Moxetumomab pasudotox-tdfk was not studied in patients with moderate to severe hepatic impairment or severe renal impairment and should not be used in patients with creatinine clearance ≤ 29 mL/min [31].

Moxetumomab pasudotox-tdfk may interfere with detection of cellular CD22; consequently, total peripheral blood B-cell counts were measured using a surrogate of CD19 + B-cell assay. Circulating CD19 + B-cells were reduced by 89% from baseline following the first three infusions of moxetumomab pasudotox-tdfk, and this was maintained for a minimum of 1 month post treatment. The B-cell count recovered approximately 6 months after the end of therapy. Additionally, a low baseline CD19 + B-cell count was found to be significantly associated with a high pharmacokinetic exposure (*P* < 0.001).

Overall, the concentration of moxetumomab pasudotox-tdfk was found to increase in proportion to the dose. The half-life of moxetumomab pasudotox-tdfk is 1.4 h with a mean estimated systemic clearance of 25 L/h after the first dose of the first cycle and decreasing to 4 L/h after subsequent doses. No systemic accumulation of moxetumomab pasudotox-tdfk was observed. Moxetumomab pasudotox-tdfk is believed to undergo proteolytic degradation into small peptides and amino acids via catabolic pathways during metabolism, but the exact mechanism is unknown [31].

## Dose preparation

It is important to understand the proper storage and preparation of moxetumomab pasudotox-tdfk. It is available as a single-dose vial containing 1 mg of preservative-free lyophilized drug powder. The IV solution stabilizer is a single-dose vial containing 1 mL of sterile, preservative-free solution, which is in a separate package from the moxetumomab pasudotox-tdfk lyophilized powder. Both the moxetumomab pasudotox-tdfk vials and the IV solution stabilizer should be stored in the refrigerator within their original cartons, protected from light, and should not be frozen. Moxetumomab pasudotox-tdfk is manufactured through a fermentation process in a nutrient medium composed of kanamycin, an aminoglycoside antibiotic. However, it should be noted that kanamycin is cleared from the manufacturing process and that it is not detectable in the final product [31].

To prepare, calculate the dose of moxetumomab pasudotox-tdfk using the patient’s actual body weight and collect the number of vials needed for the dose preparation from the refrigerator. Each vial delivers 1 mg of moxetumomab pasudotox-tdfk and is reconstituted with 1.1 mL of sterile water for injection, resulting in a concentration of 1 mg/mL. Use partial vials when necessary to avoid rounding doses down. Gently rotate or swirl for 60 s or until the powder is dissolved. Do not shake the solution [31].

The IV infusion is prepared by first adding 1 mL of the IV solution stabilizer to a 50 mL infusion bag of 0.9% sodium chloride. It is important to note that only 1 mL of IV solution stabilizer is needed to prepare a single dose of moxetumomab pasudotox-tdfk, regardless of the dose or number of vials of drug used. The infusion bag should be inverted several times to ensure that the IV solution stabilizer is adequately mixed with the sodium chloride solution. The calculated dose volume of moxetumomab pasudotox-tdfk is withdrawn from the moxetumomab pasudotox-tdfk vial(s) and is added to the sodium chloride bag containing the IV solution stabilizer. The prepared infusion bag should be gently inverted several times, but not shaken, to mix the solution. The prepared infusion bag is stable for 4 h at room temperature, or 24 h if refrigerated at 2–8 °C (36–46 °F), and should be protected from light [31]. If refrigerated, the diluted solution may be allowed to equilibrate at room temperature for a maximum of 4 h prior to administration. Reconstituted solution should not be stored and must be used immediately for dose preparation [31]. Institutional guidelines for safe storage and handling of hazardous substances should be followed.

## Administration and supportive measures

Moxetumomab pasudotox-tdfk solution is to be infused over 30 min. Prior to each infusion, patients over 50 kg should receive 1 L of hydration with an isotonic IV solution (e.g. 5% dextrose injection, 0.45% or 0.9% sodium chloride injection) over 2–4 h. For patients under 50 kg, administer 0.5 L of hydration.

Premedications are to be administered 30–90 min prior to the infusion with an antihistamine, acetaminophen, and a histamine-2 receptor antagonist.

The antihistamine and antipyretics may be continued for up to 24 h after the infusion. Additionally, an oral corticosteroid (e.g. dexamethasone 4 mg) given post infusion may decrease nausea and vomiting, both increasing tolerability and helping to maintain oral hydration and renal perfusion [47]. After the infusion, the IV line should be flushed with 0.9% sodium chloride solution to ensure that the entire dose is administered. An additional 1 L of isotonic IV solution is to be administered as post-infusion hydration, with patients under 50 kg receiving 0.5 L. In addition to IV hydration, the patient should be advised to take in up to 3 L (up to 2 L for patients under 50 kg) of oral hydration, such as water, milk, or juice, per day on days 1–8 of each cycle [31].

## Toxicities

The most common adverse reactions (with at least 20% incidence) include infusion-related reactions, edema, nausea, fatigue, headache, pyrexia, constipation, anemia, and diarrhea. Additionally, laboratory abnormalities with at least a 50% incidence include increased creatinine, increased aspartate aminotransferase and alanine aminotransferase, hypoalbuminemia, hypocalcemia, and hypophosphatemia. Laboratory abnormalities are summarized in Tables 2 and 3. Moxetumomab pasudotox-tdfk carries a boxed warning for CLS and HUS, and warnings and precautions for infusion-related reactions, renal dysfunction, and electrolyte abnormalities.

**Table 2:** Summary of laboratory abnormalities

Abnormal value	Percentage of cycles	Grade
Hypoalbuminemia	54	1–2
Elevated AST	38	1–2
Elevated ALT	38	1–2
Elevated SCr	< 10	1–2
GGT	< 10	1–3
Anemia	< 5	2
Decreased haptoglobin	< 5	3
Leukopenia	< 5	3
Thrombocytopenia	< 5	1–2
Hyperkalemia	< 5	1
Hyperuricemia	< 5	1
Hypomagnesemia	< 5	1

Phase 1 dose escalation study: 0.005–0.05 mg/kg/dose on days 1, 3, and 5; retreatment with 2–16 additional cycles (2 cycles past documentation of CR) [48]

*ALT*, alanine aminotransferase, *AST*, aspartate aminotransferase, *CR*, complete response, *SCr*, serum creatinine, *GGT*, gamma-glutamyl transferase

**Table 3:** Summary of laboratory abnormalities

Abnormal value	Percentage of patients of patients	Grades 3/4, %
Hypocalcemia	23.8	0
Hypophosphatemia	23.8	10.0
Anemia	21.3	10.0
Elevated ALT	21.3	1.3
Decreased lymphocyte count	20.0	20.0
Hypoalbuminemia	20.0	0
Hypokalemia	16.3	2.5
Hyponatremia	11.3	2.5
Neutropenia	5.0	5.0
Decreased white blood cell count	10.0	8.8

Phase 3 study: 0.04 mg/kg/dose on days 1, 3, and 5 for six cycles (maximum six cycles or until documentation of minimal residual disease-negative complete response, progression, or toxicity) [47]

*ALT*, alanine aminotransferase

## Hemolytic uremic syndrome

HUS is characterized by acute kidney injury, thrombocytopenia, and microangiopathic hemolytic anemia. HUS occurred in 7% of patients treated with moxetumomab pasudotox-tdfk in the combined safety database, including grade 3 in 3% and grade 4 in 0.8% of patients [31]. In the phase I trial, grade 2 HUS, defined as anemia with schistocytes and elevated creatinine, developed in two patients (7%) [45]. In this trial, one patient experienced thrombocytopenia with a platelet nadir of 120,000/µL on day 11 of cycle 3. The second patient had a platelet nadir of 106,000/µL on day 10 of cycle 5. The doses administered were 0.03 mg/kg/dose and 0.05 mg/kg/dose, respectively. Laboratory abnormalities also included increased creatinine (1.53 mg/dL and 1.66 mg/dL), lactate dehydrogenase (326 U/L and 238 U/L), and bilirubin (1.3 mg/dL and 1.0 mg/dL). In addition, the patient receiving 0.03 mg/kg/dose had a decrease in hemoglobin to 10.1 g/dL. In the pivotal phase 3 trial, HUS occurred in 6 patients (7.5%) receiving 0.04 mg/kg/dose [47]. Permanent discontinuation of moxetumomab pasudotox-tdfk was required in four (5.0%) of these patients. No deaths occurred as a result of HUS in prospective trials, and all laboratory abnormalities were reversible and resolved without the use of blood products or plasmapheresis. Most cases of HUS occurred in the first 9 days (range 1–16) of a treatment cycle, but cases have also been reported on other days throughout the cycle. The median time to resolution of HUS was 11.5 days (range 2–44) [31].

Although reversible and manageable, HUS is a serious treatment-related adverse event requiring vigilant monitoring and prophylaxis. Labeled guidance regarding adequate pre- and post-hydration, including oral hydration, is critical in managing fluid and electrolytes and to maintain adequate intravascular volume. Prior to each infusion, hemoglobin, platelet count, and serum creatinine should be assessed, and, if HUS is suspected, LDH, indirect bilirubin, and blood schistocytes should be assessed promptly. Thromboprophylaxis with low-dose aspirin (81 mg daily) may be considered on days 1–8 of each 28-day cycle. In the event a patient develops HUS, treatment should be discontinued [31].

## Capillary leak syndrome

CLS is a syndrome characterized by vascular permeability; signs include hypoalbuminemia, hypotension, and hemoconcentration. Although not unique to moxetumomab pasudotox-tdfk treatment, patients may experience weight gain, ascites, edema, and effusions. CLS causes an intravascular fluid and protein shift that may result in pulmonary edema, transient shock, and death. In addition to hypoalbuminemia, laboratory abnormalities include increased hematocrit, leukocytosis, and thrombocytosis.

The incidence of CLS was 34% in clinical trials, including 23, 1.6, and 2% with grades 2, 3, and 4, respectively [31]. Although most cases occurred during the first 8 days of a cycle, the median time to resolution of CLS was 12 days [31]. CLS may occur anytime throughout the cycle. For this reason, patients should be monitored for and counseled to report swelling, weight gain (greater than a 5% increase), sudden decreased blood pressure, or difficulty breathing to their healthcare provider immediately. If CLS is suspected, check oxygen saturation and evidence of pulmonary edema and/or serosal effusions.

All CLS events resolved with supportive care and/or treatment discontinuation. No deaths from CLS occurred in the clinical trials; however, the condition may be life-threatening or fatal without proper recognition and treatment. Patients with CLS have intravascular depletion, and judicious use of IV fluids is warranted. Repletion should be gradual, and use of diuretics is not recommended unless the patient is experiencing adverse events related to fluid overload. Other supportive measures include the use of IV or oral corticosteroids. Hospitalization should be considered for all patients suspected of experiencing CLS [31].

## Infusion-related reactions

Infusion-related reactions (IRRs) are a common adverse effect of many immunotherapeutic agents [52]. Despite adequate premedication with an antihistamine, acetaminophen, and a histamine-2 receptor antagonist, reactions may still occur. Overall, 50% of patients experienced some form of reaction in the phase 3 trial. Infusion reactions manifested as nausea (15%), fever (14%), chills (14%), vomiting (11%), and headache (9%) [31]. In the event of a severe reaction, the infusion should be stopped, and appropriate supportive care should be initiated. If a patient does experience an IRR, a corticosteroid should be administered prior to restarting the infusion and added as a premedication with subsequent infusions [31].

## Renal toxicity

In total, 26% of patients treated with moxetumomab pasudotox-tdfk experienced renal toxicity, which included acute kidney injury, renal failure, renal impairment, proteinuria, and increased serum creatinine [31]. Serum creatinine was found to increase by two or more grades from baseline (22% of patients), with 1.6% experiencing a grade 3 increase (greater than 3 times baseline or the upper limit of normal). Five percent of patients experienced a continued elevated serum creatinine 1.5–3 times the upper limit of normal at the end of therapy. Patients older than 65 years, those with pre-existing renal impairment, or those who experienced HUS were at an increased risk of worsening renal function from moxetumomab pasudotox-tdfk. Therapy should be delayed for grade 2 or higher increase (greater than 1.5 times baseline or upper limit of normal) in serum creatinine in patients who have a baseline serum creatinine that is within normal limits. In patients with baseline serum creatinine of grades 1–2, therapy should be delayed for any serum creatinine increase to grade 3 or higher. Moxetumomab pasudotox-tdfk may be restarted once renal function recovers to baseline or better [31].

## Electrolyte abnormalities

Overall, 57% of patients experienced electrolyte abnormalities. A summary of laboratory abnormalities from the clinical trials is provided in Tables 2 and 3. The most common abnormality was hypocalcemia, occurring in 25% of patients overall. Grade 3 and 4 electrolyte abnormalities occurred in 14% and 0.8% of patients, respectively. These abnormalities were found to occur during the same treatment cycle as other adverse effects such as CLS, HUS, fluid retention, or renal toxicity just over one-third of the time [31].

## Laboratory monitoring and vital signs

It is important to monitor blood pressure and weight at each visit. Close monitoring of complete blood count and chemistries is recommended prior to and during each cycle of moxetumomab pasudotox-tdfk. Any incidence of anemia, increased creatinine, or thrombocytopenia should be suspected as HUS. If positive, monitoring of indirect bilirubin, LDH, and a peripheral blood smear for schistocytes is warranted. Additionally, patients should be monitored for hypoalbuminemia, weight gain > 5%, hypotension unresponsive to fluids, edema, and shortness of breath, because these can be signs of CLS. If CLS is suspected, check oxygen saturations, and evaluate for pulmonary edema. Although patients may experience generalized edema as a result of moxetumomab pasudotox-tdfk, caution is advised with the use of diuretics in a patient experiencing CLS. Electrolytes should be monitored prior to each infusion and on day 8 of therapy. It is also prudent to monitor electrolytes midway through the cycle. Renal function should be monitored prior to each infusion of moxetumomab pasudotox-tdfk.

## Conclusion

Moxetumomab pasudotox-tdfk is the first FDA-approved therapy for R/R HCL, and the first new drug approved for HCL since cladribine in 1993. Patients who are no longer responding to alternative therapies or who may not tolerate further treatment with purine analogs owing to myelosuppression or infection may be good candidates for this agent. Moxetumomab pasudotox-tdfk has yielded a durable CR rate of 30% with a median duration in response that has not yet been reached as of a median follow-up of 16.7 months. The overall response rate was 75%, including 41% achieving CR and 34% achieving partial response.

Although each infusion of moxetumomab pasudotox-tdfk is relatively short at 30 min, patients must receive adequate hydration and premedication with an antihistamine, acetaminophen, and a histamine-2 receptor antagonist to prevent IRRs, HUS, and CLS. To ensure safe and effective use of moxetumomab pasudotox-tdfk, pharmacists and other providers must be vigilant in assessing for these potential events, as well as renal dysfunction and electrolyte abnormalities, and must understand proper methods for the preparation and administration of moxetumomab pasudotox-tdfk.

## Abbreviations

BCR: B-cell receptor
CLS: Capillary leak syndrome
CR: Complete response
FDA: Food and drug administration
HCL: Hairy cell leukemia
HUS: Hemolytic uremic syndrome
IRR: Infusion-related reactions
MRD: Minimal residual disease
